# Ablation of microglia does not alter circadian rhythm of locomotor activity

**DOI:** 10.1186/s13041-023-01021-1

**Published:** 2023-04-07

**Authors:** Futaba Matsui, Sho T. Yamaguchi, Riho Kobayashi, Shiho Ito, Sakimi Nagashima, Zhiwen Zhou, Hiroaki Norimoto

**Affiliations:** Graduate School of Medicine, Hokkaido University, Sapporo, Japan

**Keywords:** Microglia, Circadian rhythm, Suprachiasmatic nucleus

## Abstract

Microglia, as macrophages in the brain, are responsible for immune responses and synaptic remodeling. Although the function of microglia is regulated by circadian rhythms, it is still unclear whether microglia are involved in the generation and light entrainment of circadian rhythms of behavior. Here, we report that microglial depletion does not alter behavioral circadian rhythms. We depleted ~ 95% of microglia in the mouse brain by PLX3397, a CSF1R inhibitor, and analyzed the effect on the spontaneous behaviors of mice. We found that neither the free-running period under constant darkness nor light entrainment under jet-lag circumstances were influenced by the ablation of microglia. Our results demonstrate that the circadian rhythms of locomotor activity, an important output of the circadian clock in the brain, are likely a phenomenon not produced by microglia.

**Supplementary Information:**

The online version contains supplementary material available at 10.1186/s13041-023-01021-1.

## Introduction

Microglia are residential immune cells in the central nervous system. They are involved in the clearance of apoptotic cells and synaptic remodeling through phagocytosis [1]. Recent studies have shown that microglia are closely involved in sleep-wake cycles. For example, depletion of microglia resulted in the increase of slow-wave sleep (SWS) duration and reduced excitatory neurotransmission in the dark period [2, 3]. Another study has reported that microglial depletion suppresses rebound SWS after sleep deprivation treatment [4]. Although these studies are partially inconsistent, the results suggest a key role for microglia in the sleep/wake homeostasis.

Microglia also have circadian rhythm-related functions. Multiple clock genes and immune activation markers are expressed in roughly 24-hour cycles [5]. Furthermore, microglia exhibit circadian rhythm-dependent responses to inflammatory stimuli [6].

Thus, it is clear that microglia have a close association with circadian rhythms as neurons and astrocytes; however, the evidence supporting the roles of microglia on circadian clock system itself, particularly on the locomotor activity rhythms, has still been inconclusive [7, 8, 9]. We re-examined this issue by using the CSF1R inhibitor PLX3397 treated mice. Microglia in the adult brain are fully dependent upon CSF1R signaling for their survival. PLX3397 can be administered through food chow with minimal behavioral interference to achieve robust microglial elimination, and so far, no effects on typical animal behavior and cognitive functions have been reported [10]. Therefore, it is an ideal way to examine the role of microglia in spontaneous animal behavior [11].

## Results & discussions

**Fig. 1:**
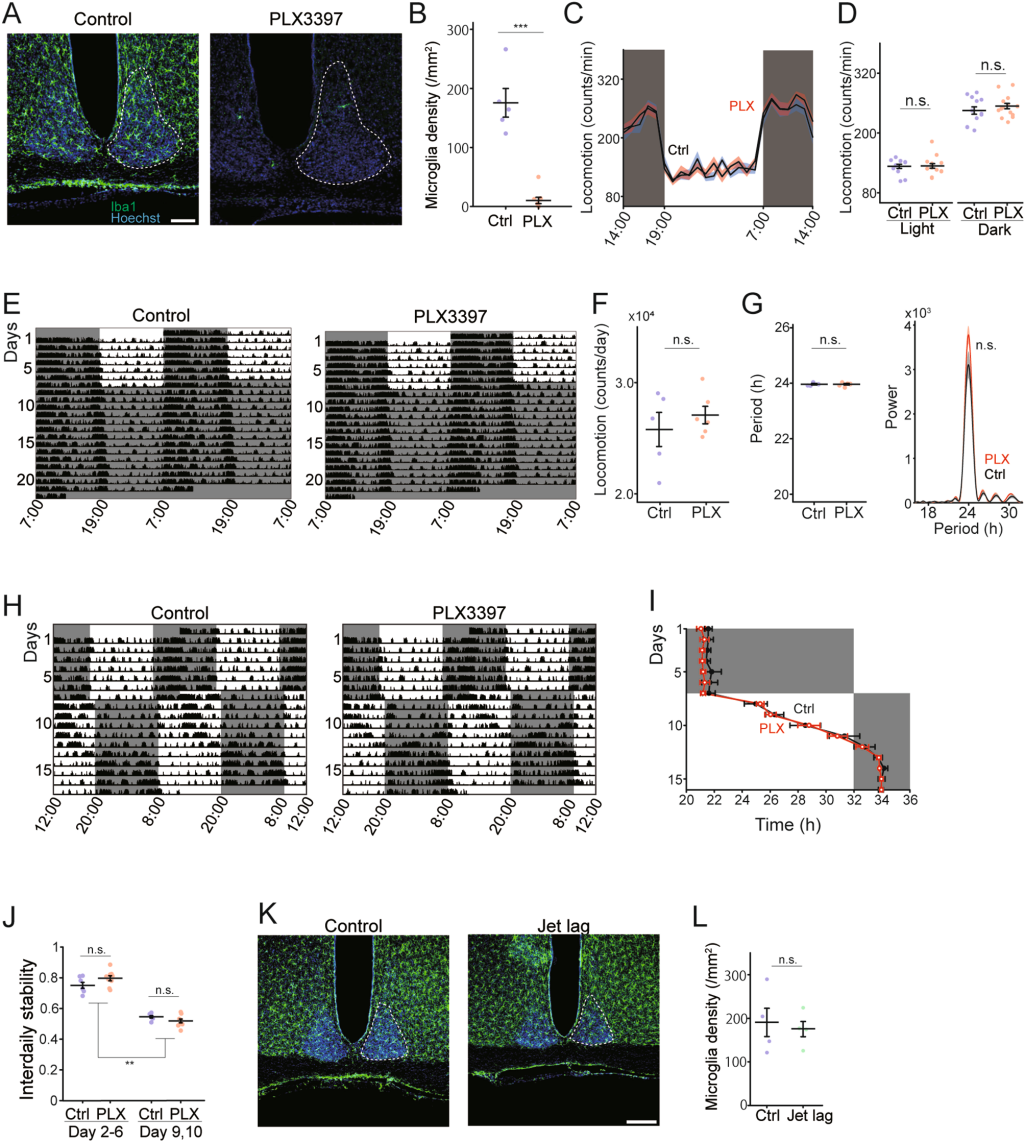
(A) Coronal brain sections from a control mouse (Left) and PLX3397 treated mouse (Right) with Hoechst staining (blue) and microglial marker IBA1 (green) immunolabeling. The dotted line indicates the SCN. Scale bar = 100 μm. (B) PLX3397 treatment induced a 95% microglial depletion in the SCN. N = 7 mice (Control), 8 mice (PLX3397). ***P < 0.001. (C) Mean activity profiles were generated from 7 days in 12 L:12D. N = 12 (Control) and 15 (PLX3397) mice. (D) Analyses of day and night spontaneous locomotor activity counts. (E) Representative double-plotted actograms of control and PLX3397 mice in DD. Shaded gray areas in the actogram represent dark periods. (F) Analyses of spontaneous locomotor activity counts in DD. N = 5 (Control) and 6 (PLX3397) mice. (G) Estimated periods (left) and power (right) of circadian rhythms by the Lomb-Scargle periodogram. N = 5 (Control) and 6 (PLX3397) mice. (H) Representative double-plotted actograms of control and PLX3397 mice subjected to a 13-hour phase advance in LD cycles. (I) Activity onset in the 13-hour phase advance. N = 6 (Control) and 9 (PLX3397) mice. (J) Average interdaily stability from Day 2 to Day 6 (before jet-lag) and from Day 9 to Day 10 (during jet-lag). N = 6 (Control) and 9 (PLX3397) mice. **P < 0.01. (K) Coronal brain section from a control mouse (Left) and a mouse under jet-lag condition (Right) with Hoechst (blue) and microglial marker IBA1 (green) immunolabeling. Scale bar = 100 μm. (L) SCN microglial density from the control group and jet-lag group. N = 4 mice for each group. Data are presented as means ± SEM with individual data points plotted. n.s.= non-significant difference.

Mice were randomly assigned to two groups fed with either a rodent standard chow (control) or chow with a CSF1R inhibitor PLX3397, for three weeks. The number of microglia in the suprachiasmatic nucleus (SCN), which is well known to generate circadian rhythms, was significantly reduced in the PLX3397 group (Fig. 1A, B, *P* = 2.8 × 10^− 7^, *t*_14_ = 9.14, Student’s *t*-test). Microglia depletion was also observed in other brain regions, consistent with previous reports (data not shown) [10]. No difference in locomotor activity was observed between the control and PLX3397 groups (Fig. 1C, D, *P* = 0.89, *t*_25_= -0.15 (Light), *P* = 0.35, *t*_25_ = -0.95 (Dark), Student’s *t*-test).

To examine the effect of microglia depletion on behavioral circadian rhythms, we measured animals’ free-running rhythms under constant darkness (DD) condition. The mice were housed in a light controlled home cage with access to food chow and water *ad libitum*, and their spontaneous locomotor activity was recorded by accelerometers. The animals had been receiving the PLX3397 treatment for three weeks on day 1 of behavior monitoring. The mice were housed in a 12-hour light/12-hour dark (LD) cycle for one week and then placed in a DD condition for two weeks. Both groups exhibited robust rhythms of free-running locomotor activity, and the total locomotor activity did not differ between the control and PLX3397 treated mice during DD condition (Fig. 1E, F, *P* = 0.45, *t*_9_ = -0.79, Student’s *t*-test). The free-running periods and the power calculated by Lomb-Scargle periodogram also did not differ between the two groups (Fig. 1G, *P* = 0.99, *t*_9_= -0.02 (periods), *P* = 0.114, *t*_9_= -0.1139 (power), Student’s *t*-test).

These results imply that microglia do not affect the internal autonomous clock, but it is still possible that microglia function during light entrainment. To test the possibility, we examined the effect of microglia depletion on behavioral rhythms under experimental jet-lag conditions. After 1 week of recording behaviors in a normal LD condition, LD cycles were advanced by 13 h (Fig. 1H). In both groups, this advance of LD cycles induced a gradual shift of locomotor activity rhythms, which took 5–6 days for the complete re-entrainment to the new LD schedule (Fig. 1H, I). Interdaily stability, a measure of the strength of circadian rhythmicity, was reduced during the re-entrainment to the new LD cycles (Fig. 1J, Control: *P* = 1.0 × 10^− 3^, *Q*_6,6_ =12.87, Day 2–6 vs. Day 9,10, PLX: *P* = 1.0 × 10^− 3^, *Q*_8,8_ =19.78, Day 2–6 vs. Day 9,10, Tukey test after one-way ANOVA). However, both the onset timing and the interdaily stability were similar between control and microglia depletion groups (Fig. 1I, *P* = 0.17, *F*_1,1_ = 2.08, repeated measure two-way ANOVA; Fig. 1J, *P* = 0.59, *Q*_6,8_ =1.78, Day 9, 10, Control vs. PLX, *P* = 0.16, *Q*_6,8_ =3.08, Day 2–6, Control vs. PLX, Tukey test after one-way ANOVA). Finally, we examined the changes in the microglia density after experiencing the jet-lag. Mice were perfused after three days of LD cycle advancement and immunostained for microglia marker IBA1. There was no difference in microglial density between control and jet-lag group (Fig. 1K, L, *P* = 0.73, *t*_6_ = 0.36, Student’s *t*-test).

In the present study, we demonstrated that microglial depletion does not affect the daily locomotor activity, free-running rhythms in a DD condition, and the light entrainment of activity rhythms. These results are in a way unexpected because microglia regulate not only higher order brain function in the forebrain but also hypothalamic circuits via the release of inflammatory factors and dynamic remodeling of synapses [12]. It should be noted that these findings are in marked contrast with previous reports showing that microglial ablation partially disrupts the circadian system using Cx3cr1-Dtr transgenic rats [8]. The contradiction may be explained by survival rate of microglia, alternative off-target effects of the ablation method [13], or distinct functional states of microglia in the different experimental conditions. Further investigations using functional imaging techniques and manipulating microglial activity *in vivo* will help resolve the current controversy regarding the function of microglia on circadian rhythms.

## Electronic supplementary material

Below is the link to the electronic supplementary material.

**Additional file 1**: Detailed methods.

## Data Availability

The datasets used and/or analyzed for the current study are available from the corresponding author upon request.

## Abbreviations

SCN: Suprachiasmatic nucleus
CSF1R: Colony stimulating factor 1 receptor
SWS: Slow wave sleep
LD: Light/dark
DD: Constant darkness
