# Supplementary material for: Ablation of microglia does not alter circadian rhythm of locomotor activity
Source: Mol Brain. 2023 Apr 7;16:34. doi: 10.1186/s13041-023-01021-1 (PMC10080745; doi:10.1186/s13041-023-01021-1)
Supplement: Supplementary file 1 — Additional file 1: Detailed methods. [file 13041_2023_1021_MOESM1_ESM.docx]

**Additional file 1**

Ablation of microglia does not alter circadian rhythm of locomotor activity

Futaba Matsui^1^, Sho T. Yamaguchi^1^, Riho Kobayashi^1^, Shiho Ito^1^, Sakimi Nagashima^1^, Zhiwen Zhou^1,†^, Hiroaki Norimoto^1,†^

^1^ Graduate School of Medicine, Hokkaido University, Sapporo, Japan

†To whom correspondence should be addressed:

Hiroaki Norimoto, Ph.D.

Zhiwen Zhou, Ph.D.

Department of Cellular Pharmacology

Graduate School of Medicine, Hokkaido University

Kita 15, Nishi 7, Kita-ku, Sapporo, 060-8638, Japan

Tel: +81-11-706-6919

E-mail: norimoto@pop.med.hokudai.ac.jp

E-mail: zzhou@pop.med.hokudai.ac.jp

**Materials and Methods**

**Animals**

Male adult (6-to-9-week-old) C57BL/6J mice were housed under standard laboratory conditions (12-h light/dark cycle, free access to food and water). All efforts were made to minimize the animals' suffering and the number of animals used.

**Surgery**

Mice were anesthetized by isoflurane inhalation (Mylan Inc., Pittsburgh, PA, USA). Lidocaine (Sandoz Pharma, Tokyo, Japan) was used as local anesthesia for the incisions. After approximately 2 cm of the dorsal skin was incised, the nano tag® (Kissei Comtec, Nagano, Japan), an accelerometer, was implanted subcutaneously. Antibiotic sulfadimethoxine (Nippon Zenyaku Kogyo Co., Ltd., Fukushima, Japan) was administered at doses of 50 mg/kg the day of operation and 25 mg/kg/day for the following two days.

**PLX3397 treatment**

PLX3397 (C-1271; Chemgood, VA, USA) was mixed into standard rodent chow (Research Diets Inc, NJ , USA) at the concentration of 290 mg /kg chow to achieve a dose of approximately 46 mg/kg body weight per day for three weeks, which was found to be successful in reducing microglia in mice [1]. A total of 15 mice received the PLX diet.

**Behavior activity monitoring**

Locomotor activity was recorded in 0.5-min bins with nano tag®, and the data obtained were analyzed with ActogramJ [2] and Python. Activity onsets were defined as the intersections between a short moving average (3-h window) and a long moving average (24-h window) of the smoothed time series [3]. More specifically, for each time bin (*i*), the difference between short and long moving averages (Δ*i*) was determined. The daily onset of activity was the time point at which the short moving average first exceeded the long moving average, that is, when Δ*i* > 0 and Δ*i*−1 < 0.

Interdaily stability was derived by normalizing (for the number of data) the 24-h value from the chi-square periodogram:

*
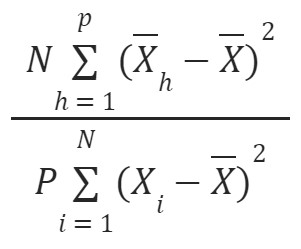
*

Interdaily stability =

where *N* is the total number of data; *p* is the number of data per day; $\bar{X}$ is the mean of all data; $\bar{X}_{h}$ are the hourly means; $X_{i}$ represent the individual data points. In this study, interdaily stability was calculated from every 3 consecutive days. For example, Interdaily stability of Day 2 was calculated from hourly activity from Day 1 to Day 3.

**Immunohistochemistry**

Animals were perfused transcardially with cold phosphate-buffered saline (PBS) followed by 4% paraformaldehyde in 0.1 M PBS, and the brains were quickly removed and placed in 4% paraformaldehyde for 24 hours and then placed in 0.1 M PBS with 30% sucrose for 48 hours at 4°C. 100-μm-thick brain sections were made using a vibratome (VT1200, Leica, Wetzlar, Germany).

The brain sections were permeabilized, and nonspecific staining was blocked with 10% goat serum (Funakoshi, Tokyo, Japan) in 0.3% Triton X-100 (TaKaRa, Shiga, Japan) in PBS. For immunohistochemical detection of IBA1, we used the primary antibody rabbit anti-IBA1 antibody (1:500, Wako, Osaka, Japan). Sections were incubated overnight at 4 °C with primary antibody. After extensive washes in PBS, the sections were incubated with the appropriate secondary antibody conjugated with Alexa Fluor dyes (1:500; Invitrogen, MA, USA). After incubation overnight at 4 °C with the secondary antibody, the sections were extensively washed with PBS and mounted on glass slides. The sections were photographed using an FV1000 confocal scanning microscope with 10×/20× objective lenses (Olympus, Tokyo, Japan). Images were processed using Fiji ImageJ (NIH, USA).

1. Elmore MR, Najafi AR, Koike MA, Dagher NN, Spangenberg EE, Rice RA, Kitazawa M, Matusow B, Nguyen H, West BL *et al*: **Colony-stimulating factor 1 receptor signaling is necessary for microglia viability, unmasking a microglia progenitor cell in the adult brain**. *Neuron* 2014, **82**(2):380-397.

2. Schmid B, Helfrich-Forster C, Yoshii T: **A new ImageJ plug-in "ActogramJ" for chronobiological analyses**. *J Biol Rhythms* 2011, **26**(5):464-467.

3. van der Vinne V, Riede SJ, Gorter JA, Eijer WG, Sellix MT, Menaker M, Daan S, Pilorz V, Hut RA: **Cold and hunger induce diurnality in a nocturnal mammal**. *Proc Natl Acad Sci U S A* 2014, **111**(42):15256-15260.
